# LORETA Current Source Density for Duration Mismatch Negativity and Neuropsychological Assessment in Early Schizophrenia

**DOI:** 10.1371/journal.pone.0061152

**Published:** 2013-04-05

**Authors:** Tomohiro Miyanishi, Tomiki Sumiyoshi, Yuko Higuchi, Tomonori Seo, Michio Suzuki

**Affiliations:** Department of Neuropsychiatry, University of Toyama Graduate School of Medicine and Pharmaceutical Sciences, Toyama, Japan; Chiba University Center for Forensic Mental Health, Japan

## Abstract

**Introduction:**

Patients with schizophrenia elicit cognitive decline from the early phase of the illness. Mismatch negativity (MMN) has been shown to be associated with cognitive function. We investigated the current source density of duration mismatch negativity (dMMN), by using low-resolution brain electromagnetic tomography (LORETA), and neuropsychological performance in subjects with early schizophrenia.

**Methods:**

Data were obtained from 20 patients meeting DSM-IV criteria for schizophrenia or schizophreniform disorder, and 20 healthy control (HC) subjects. An auditory odd-ball paradigm was used to measure dMMN. Neuropsychological performance was evaluated by the brief assessment of cognition in schizophrenia Japanese version (BACS-J).

**Results:**

Patients showed smaller dMMN amplitudes than those in the HC subjects. LORETA current density for dMMN was significantly lower in patients compared to HC subjects, especially in the temporal lobes. dMMN current density in the frontal lobe was positively correlated with working memory performance in patients.

**Conclusions:**

This is the first study to identify brain regions showing smaller dMMN current density in early schizophrenia. Further, poor working memory was associated with decreased dMMN current density in patients. These results are likely to help understand the neural basis for cognitive impairment of schizophrenia.

## Introduction

Schizophrenia is a chronic and progressive psychotic disorder that emerges mainly in late adolescence or early adulthood. Patients with the illness exhibit positive symptoms and negative symptoms, as well as disturbances of various domains of cognitive function, e.g. verbal memory, working memory, executive function, and attention [1], [2]. In particular, cognitive impairments have been shown to disturb their social activities, work outcome, and quality of life. Recent studies [3], [4] report that mild cognitive deficits already exist before the onset of schizophrenia, or “at risk mental state” (ARMS). The neural substrates for cognitive deficits may include some brain regions, such as hippocampus and parahippocampal gyrus [5]–[8].

Mismatch negativity (MMN) is one of the event-related potentials (ERPs) generated by a deviant (infrequent) stimulus. MMN is elicited even under pre-attentive conditions, and reflects an automatic pre-attention process. Generation of the MMN is an indicator of auditory sensory memory, and represent information processing dependent on some components of the auditory cortex, e.g. superior temporal gyrus [9]–[11]. Previous studies using low-resolution brain electromagnetic tomography (LORETA), fMRI, and other procedures have demonstrated that MMN reflects activities of a neural network involving several brain structures. Among them, the auditory cortex plays a key role in the complex neural architecture of sensory discrimination [12]–[14].

The feature of MMN waveforms varies according to type of deviant stimuli, i.e. frequency, duration, intensity, and location. For example, diminished MMN amplitudes reflect cognitive decline in psychiatric conditions [15]. In schizophrenia, smaller amplitudes of MMN, especially duration MMN (dMMN), have been reported [3], [16]–[19].

Several attempts have been made to relate MMN amplitudes and neuropsychological performance [17]–[21]. Lin et al. used predictive multivariate logistic regression model, and demonstrated dMMN and performance IQ, evaluated by the Wechsler Adult Intelligence Scale-Third Edition, can distinguish between schizophrenia patients and healthy control (HC) subjects [22].

LORETA provides three-dimensional images of brain electrical activity [23]. There are only a few reports on LORETA analysis of MMN in schizophrenia. Park et al. (2002) [24] observed a significant decrease in the current density for frequency MMN in the left superior temporal gyrus and left inferior parietal gyrus in patients with schizophrenia. Recently, Takahashi et al (2013) report reduced dMMN current density at right medial frontal gyrus, right cingulate gyrus, and right paracentral lobule in patients with chronic schizophrenia [12]. To our knowledge, there is no report on LORETA analysis of dMMN in early psychosis.

The above considerations indicate the ability of the combination of neuropsychological tests and dMMN to provide an objective measure to diagnose schizophrenia. So far, no study has investigated the correlation between MMN current density in some brain regions, e.g. frontal lobe, and neuropsychological performance.

Therefore, this study was conducted to test the hypotheses that 1) patients with schizophrenia would exhibit decreased dMMN current density in brain areas relevant to the pathophysiology of the illness, such as some temporal lobe structures [25], and 2) reduced dMMN current density in the frontal lobe would be associated with impairment of neuropsychological performance, such as working memory.

## Methods

### Ethics Statement

This protocol was approved by the Committee on Medical Ethics of the University of Toyama. After a complete and detail description of the study was given, subjects provided written informed consent. Clinical staff explained the nature of the study to the subjects, the risks and benefits, and the option not to participate in this research. If the mental status of a subject was impaired to the point where s/he could not understand these issues, the subject was not asked to participate in this research. If there was a possibility that the capacity of a participant to consent was compromised, an additional consent form was obtained from the next of kin, care takers, or guardians of such subjects.

### Participants

Subjects were diagnosed by experienced psychiatrists, based on the Structured Clinical Interview for DSM-IV (SCID) for schizophrenia or schizophreniform disorder. Twenty patients (male/female, 9/11; mean [S.D.] age, 27.2 [7.3]) participated in this study. Their duration of illness was less than 2 years. Twenty HC participants (male/female, 14/6; mean [S.D.] age, 25.4 [6.9]) were also recruited. They had no personal history of psychiatric illnesses, including schizophrenia and other psychotic disorders. All participants were right-handed. Psychiatric and treatment histories were obtained from the subjects, family members, and medical records. Subjects with a current history of substance abuse or dependence, seizure, or head injury were excluded from the study. Complete physical examination revealed no neurological illness for all subjects. Demographic data at baseline evaluation are shown in Table 1.

**Table 1:** Demographic and clinical data.

	Healthy	Early
	controls	schizophrenia
	(n = 20)	(n = 20)
Male/Female	14/6	9/11
Age (years)	25.4 (6.9)	27.2 (7.3)
	range, 16–45	range, 16–38
Education (years)	15.1 (2.9)	13.2 (2.1) *
Age at onset (years)	-	26.5 (7.1)
Duration of illness (years)	-	0.6 (0.5)
Antipsychotic dose	-	2.1 (2.4)
(Risperidone equivalent mg/day)		
SAPS	-	16.5 (13.2)
SANS	-	53.9 (25.2)
BACS-J (Z-score)#		
Verbal memory		−1.22 (1.59)
Working memory		−1.16 (1.18)
Motor function		−2.52 (1.07)
Verbal fluency		−1.12 (0.77)
Attention		−1.65 (0.75)
Executive function		−0.40 (1.89)

Values represent means (SD).

SAPS, Scale for the Assessment of Positive Symptoms.

SANS, Scale for the Assessment of Negative Symptoms.

BACS-J, Brief Assessment of Cognition in Schizophrenia, Japanese version.

* p<0.05, significantly smaller than healthy controls.

# SD unit compared to reported values (ref. [27], [28]).

### Clinical and neurocognitive assessment

The Scale for the Assessment of Positive Symptoms (SAPS) and the Scale for the Assessment of Negative Symptoms (SANS) [26] were administered by an experienced psychiatrist. These data are shown in Table 1.

Neuropsychological performance, measured by the brief assessment of cognition in schizophrenia Japanese version (BACS-J) [27], was evaluated by experienced psychiatrists or psychologists. The BACS-J uses the following assessments in the respective targeted domains; list learning (verbal memory), digit sequencing task (working memory), token motor task (motor function), category fluency and letter fluency (verbal fluency), symbol coding (attention and processing speed), and the Tower of London test (executive function) [27], as shown in Table 1. These scores were transformed into Z-scores using data from healthy volunteers, as previously reported [27], [28]. Raters were not informed of subjects' profiles or their diagnoses.

### Electroencephalographic recording

Electroencephalograms (EEGs) were recorded based on previous reports from our laboratory [29]–[34]. A 32-channel DC-amplifier (EEG-2100 version 2.22J, Nihon Kohden Corp., Tokyo, Japan), according to the international 10–20 system, was used. Recordings were performed using an electro cap (Electro cap Inc., Eaton, OH) in a sound-attenuated room. Data were collected with a sampling rate of 500 Hz. All electrodes were referred to the average amplitude of the ear electrodes (bandwidth, 0.53–120 Hz, 60 Hz notch filter). Electrode impedance was less than 5 kΩ. Measurements of dMMN were based on our previous report [33]. One thousand auditory stimuli were delivered binaurally through headphones with inter-stimulus intervals of 500 ms. Standard/target tones of 50/100 ms duration were randomly presented with a presentation probability of 0.9/0.1. All tones were 60 dB, 1000 Hz and with a rise-fall time of 10 ms. Subjects were requested to watch a silent animated movie (Tom and Jerry®), and to pay attention to the monitor and ignore the tones. Averaging of ERP waves and related procedures were performed using Vital Tracer and EPLYZER II software (Kissei Comtec, Co. Ltd. Nagano, Japan). Epochs were 600 ms, including a 100-ms pre-stimulus baseline. Eye movement artifacts (blinks and eye movements) were manually rejected. MMN waveforms were obtained by subtracting the standard waveforms from the target waveforms. ERP component peaks were identified within the fixed search windows between 100–250 ms. We confirmed the presence of the peaks of MMN in all subjects.

### LORETA analysis

LORETA images were obtained by estimating the current source density distribution for epochs of brain electric activity on a dense grid of 2394 voxels at 7-mm spatial resolution applied to the digitized Talairach and Tournoux (1988) [35], based on the established method [23]. LORETA made use of the three-shell spherical head model registered to the Talairach atlas available as a digitized MRI from the Brain Imaging Centre, Montreal Neurologic Institute. Registration between spherical and realistic head geometry used EEG electrode coordinates reported by Towle et al (1993) [36]. The solution space was restricted to cortical gray matter and the hippocampus, as determined by the corresponding digitized Probability Atlas also available from the Brain Imaging Centre. A voxel was labeled as gray matter if it met the following three conditions: its probability of being gray matter was higher than that of being white matter, its probability of being gray matter was higher than that of being cerebrospinal fluid, and its probability of being gray matter was higher than 33% [23]. We used the original LORETA version reported by Pascual-Marqui et al [23]. We calculated LORETA images for each subject in the fixed time frame between the 100–250 ms post-stimulus period to obtain the LORETA value for each voxel. Additionally, we averaged LORETA value containing the following brain regions of interest (ROI): frontal lobe, temporal lobe, parietal lobe, and occipital lobe.

### Data analysis

Statistical analyses were performed using the Statistical Package for Social Sciences (SPSS) version 20 (SPSS Japan Inc., Tokyo, Japan). To investigate differences between groups, dMMN amplitudes at the Fz lead were assessed by independent t-test. Comparisons between early schizophrenia and HC on LORETA source imaging were conducted using voxel-by-voxel unpaired *t*-statistics after logarithmic transformation of the data. Holmes' non-parametric correction for multiple comparisons was applied [37]. Relationships of LORETA current density with BACS-J domain scores, SAPS total scores, and SANS total scores were analyzed using Spearman rank correlations. Bonferroni correction was applied for multiple comparisons. LORETA current density for dMMN did not show a uniformly normal distribution. Therefore, dMMN current density was subjected to natural logarithmic transformation to obtain a more normal distribution. The significance level for all statistical tests was set at p<0.05 (two-tailed).

## Results

### Subjects' profiles

Demographic data of participants are shown in Table 1. The female to male ratio and age were not significant between patients and HC (data not presented). Education level was significantly lower in patients than in HC subjects (t = 2.29; p = 0.028).

### Neuropsychological assessments

BACS data for patients are shown in Table 1. Except for executive function, the Z-scores of the other domains were below −1.0. Especially motor function was severely impaired.

### Comparisons of dMMN amplitudes between HC and early schizophrenia

**Figure 1:**
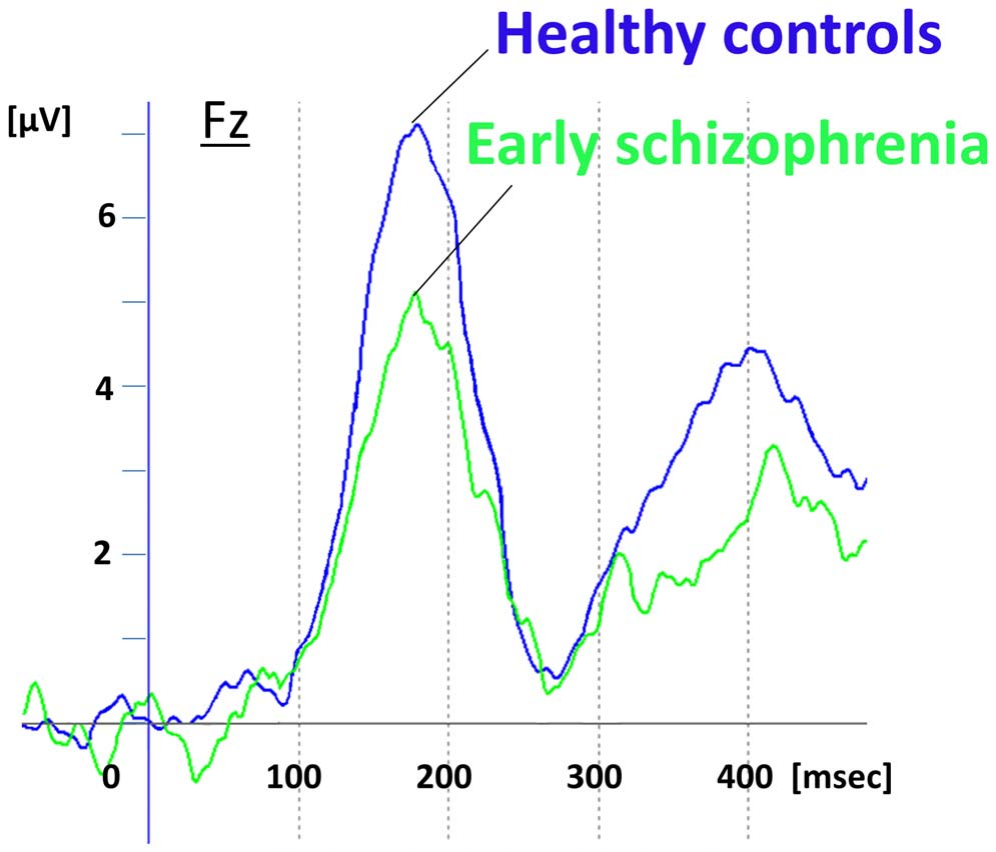
Duration mismatch negativity (dMMN) waveforms at the Fz lead. dMMN waveforms for healthy controls (N = 20, blue line) and early schizophrenia (N = 20, light green line) are shown.

Figure 1 shows the overall average dMMN waveforms in the Fz lead. dMMN amplitudes in HC and patients (mean ± SD) were 7.9±1.1 µV and 5.6±1.7 µV, respectively. Patients showed significantly smaller dMMN amplitudes than did HC subjects (t = 4.97; p<0.01).

### Comparison of LORETA images for dMMN between HC and early schizophrenia

**Figure 2:**
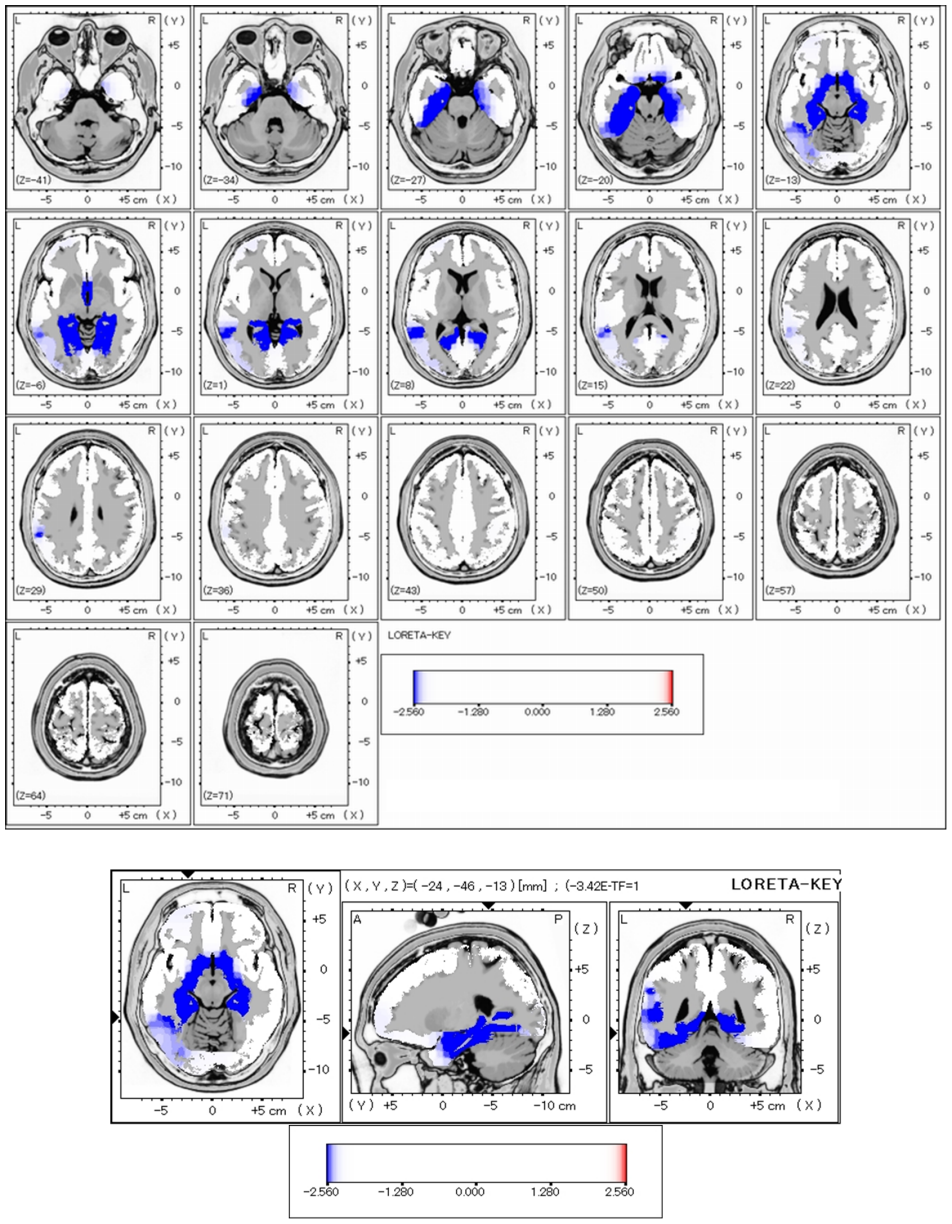
LORETA images for dMMN. Comparison of LORETA current density for dMMN between early schizophrenia (N = 20) and healthy control (N = 20, HC) subjects, as revealed by statistical non-parametric mapping voxel-wise comparison for independent samples. Blue areas represent brain regions showing significantly lower LORETA values for early schizophrenia subjects in comparison with HC subjects.

We compared LORETA current source density of dMMN between HC and early schizophrenia. Compared to HC subjects, patients elicited a significantly lower current density in several brain regions, especially those in the temporal lobes, such as parahippocampal gyrus and hippocampus (Figure 2). Additionally, dMMN current density in the frontal structures, such as anterior cingulate, was significantly lower for early schizophrenia. Table 2 demonstrates brain areas showing the largest difference in dMMN current density.

**Table 2:** Coordinates for brain areas showing the largest differences (top five) between healthy controls and early schizophrenia in dMMN current density.

		(X,Y,Z)	P-value
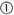	left parahippocampal gyrus	−24, −46, −13	<0.01
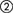	left fusiform gyrus	−31, −46, −6	<0.01
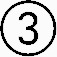	right parahippocampal gyrus	11, −39, 1	<0.05
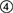	right hippocampus	25, −39, 1	<0.05
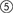	left anterior cingulate	−3, −11, −6	<0.05

### Relationship between psychotic symptoms and LORETA current density for dMMN

There was no significant correlation between the SAPS or SANS score vs. LORETA current density for dMMN in any brain region (data not presented).

### Relationship between neuropsychological assessment and dMMN current density

**Figure 3:**
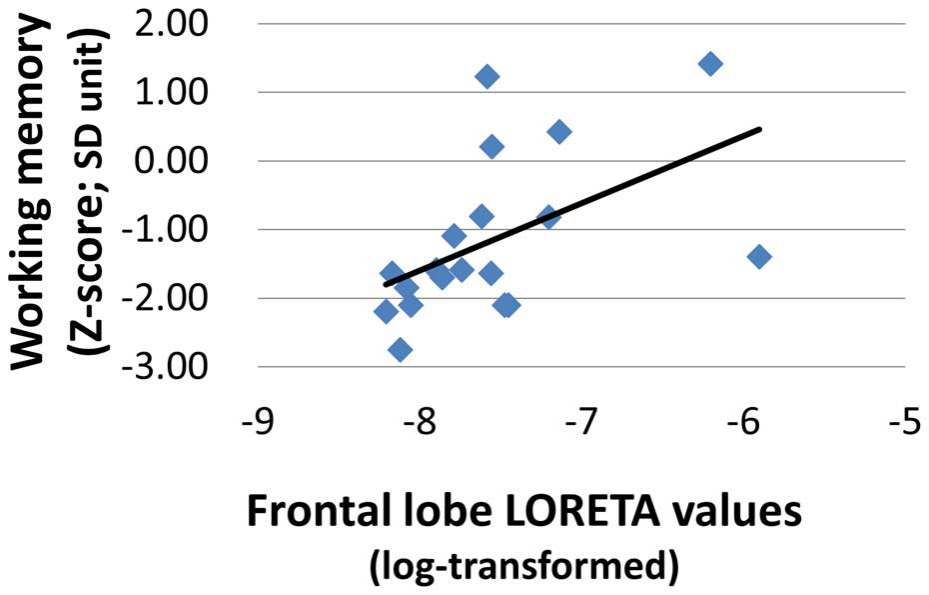
Correlations between dMMN current density and working memory. Scatterplots and least squares regression lines are shown for the correlations between LORETA current density for dMMN (log-transformed) and neuropsychological performance in early schizophrenia.

Table 3 demonstrates the relationships between BACS-J domain scores and LORETA current density for dMMN. dMMN current density in the frontal lobe was positively correlated with working memory in patients with early schizophrenia (Table 3, Figure 3). The correlation remained significant even after Bonferroni correction was applied. There were no such correlations for temporal, parietal, and occipital lobes.

**Table 3:** Spearman correlation coefficients between dMMN current density (log-transformed) in discrete brain regions and BACS-J scores in early schizophrenia.

	verbal		working		motor		verbal		attention		executive	
	memory		memory		function		fluency				function	
	r	P	r	P	r	P	r	P	r	P	r	P
Frontal lobe	0.308	0.199	0.587	**0.008**	−0.102	0.678	−0.275	0.254	0.097	0.691	−0.092	0.707
Temporal lobe	0.259	0.285	0.448	0.055	−0.220	0.366.	−0.244	0.314	0.116	0.637	−0.108	0.659
Parietal lobe	0.110	0.655	0.274	0.257	−0.146	0.551	−0.256	0.290	0.150	0.540	−0.221	0.364
Occipital lobe	0.072	0.770	0.336	0.160	−0.100	0.683	0.107	0.663	0.372	0.117	0.028	0.909

## Discussion

To our knowledge, this is the first study to report three-dimensional distribution patterns of dMMN current density and neuropsychological performance in early schizophrenia in comparison with healthy controls. LORETA images demonstrated a decreased dMMN current density in brain areas known to be associated with the pathophysiology of the illness, e.g. parahippocampal gyrus, hippocampus, fusiform gyrus, and anterior cingulate [5], [6], [38], [39]. We also observed positive correlations between dMMN current density in the frontal lobe and working memory performance in patients with early schizophrenia.

Reductions in the volume of several brain regions, including frontal cortex and temporal cortex, in schizophrenia subjects and individuals vulnerable to developing the illness have been reported [40]–[42]. Reduced dMMN current density in the temporal lobe of patients, observed in this study, is consistent with these morphological findings. Specifically, MMN has been considered to be generated by neural activities in the superior temporal cortex and frontal cortex [9]–[11]. The present data from a more feasible and non-invasive methodology (i.e. EEG) add support to these lines of evidence for the potential role of several discrete brain regions in the pathophysiology of schizophrenia.

Takahashi et al. (2013) report schizophrenia patients demonstrated a smaller dMMN current density in the right medial frontal gyrus [12]. Compared with our data, their results indicate more frontal regions are affected in patients [12]. The discrepancy may be due to the difference in duration of illness. The subjects of Takahashi's study were chronic schizophrenia, with a mean duration of illness of 23.6 years, while that of our subjects was shorter, i.e. less than 2 years. It is possible that the electrophysiological impairment, e.g. dMMN, becomes more extensive as psychosis progresses. In this context, further study is needed to examine a longitudinal course of dMMN in schizophrenia.

Correlations between MMN amplitudes and neuropsychological performance have been an issue for intensive investigations. Several [17]–[21], but not all [3], [22] studies found MMN amplitudes to be related to cognitive function. The present study revealed, for the first time, that dMMN current density in early schizophrenia was correlated with working memory. Perlstein et al. [43] report that this cognitive domain was associated with dorsolateral prefrontal cortex function, as measured by fMRI, consistent with our electrophysiological findings. Further study should clarify sub-region(s) of the frontal cortex whose dMMN current density is specifically associated with working memory.

The limitations of the present study should be noted. Patients with early schizophrenia were taking antipsychotic drugs which are agonists at dopamine receptors, although modulations of dopaminergic transmission have been shown to exert little effect on dMMN [44], [45]. Another limitation may be the use of the original version of LORETA. Further study is warranted to examine dMMN in drug-naïve subjects using an updated version of LORETA (e.g. eLORETA, sLORETA), which would be more advantageous for sub-region analyses and/or multiple comparisons.

In conclusion, this study provides, for the first time, information on the brain regions responsible for diminished dMMN amplitudes in subjects with early schizophrenia. Further, we have found associations between poor working memory and decreased dMMN current density in these patients. These results are likely to help understand the neural basis for cognitive impairment of schizophrenia.
